# Lactylation: the novel histone modification influence on gene expression, protein function, and disease

**DOI:** 10.1186/s13148-024-01682-2

**Published:** 2024-05-29

**Authors:** Yue Hu, Zhenglin He, Zongjun Li, Yihan Wang, Nan Wu, Hongyan Sun, Zilong Zhou, Qianying Hu, Xianling Cong

**Affiliations:** 1 Department of Tissues Bank, China-Japan Union Hospital of Jilin University, Changchun, 130033 China; 2 China-Japan Union Hospital of Jilin University, Jilin University, Changchun, 130033 China; 3 Department of Dermatology, China-Japan Union Hospital of Jilin University, Changchun, 130033 China

**Keywords:** Lactic acid, Lactylation, Protein posttranslational modifications, Metabolism, Disease

## Abstract

Lactic acid, traditionally considered as a metabolic waste product arising from glycolysis, has undergone a resurgence in scientific interest since the discovery of the Warburg effect in tumor cells. Numerous studies have proved that lactic acid could promote angiogenesis and impair the function of immune cells within tumor microenvironments. Nevertheless, the precise molecular mechanisms governing these biological functions remain inadequately understood. Recently, lactic acid has been found to induce a posttranslational modification, lactylation, that may offer insight into lactic acid's non-metabolic functions. Notably, the posttranslational modification of proteins by lactylation has emerged as a crucial mechanism by which lactate regulates cellular processes. This article provides an overview of the discovery of lactate acidification, outlines the potential “writers” and “erasers” responsible for protein lactylation, presents an overview of protein lactylation patterns across different organisms, and discusses the diverse physiological roles of lactylation. Besides, the article highlights the latest research progress concerning the regulatory functions of protein lactylation in pathological processes and underscores its scientific significance for future investigations.

## Introduction

Lactic acid is traditionally considered as a metabolic waste product produced during glycolysis in normal cells under hypoxic conditions. In the 1920s, Otto Warburg's observations revealed that tumor cells exhibit enhanced glucose uptake and prefer glycolysis even in the absence of hypoxia, resulting in increased lactic acid production through aerobic glycolysis, known as the Warburg effect [1, 2]. Lactic acid has garnered scientific attention due to its association with tumor metabolism. Based on the results from Warburg, recent research has uncovered the multifaceted biological functions of lactic acid, including its role as an energy source that can be transported into cells [3–5], its involvement in promoting tumor angiogenesis [6–9], its ability to inhibit immune cells within the tumor microenvironment [10–12], and its function as a signaling molecule mediating intracellular and intercellular communication. Furthermore, lactic acid has been implicated in regulating natural immune signals [13] and other related processes. However, the precise molecular mechanisms governing these diverse biological functions of lactic acid require further investigation.

**Fig. 1:**
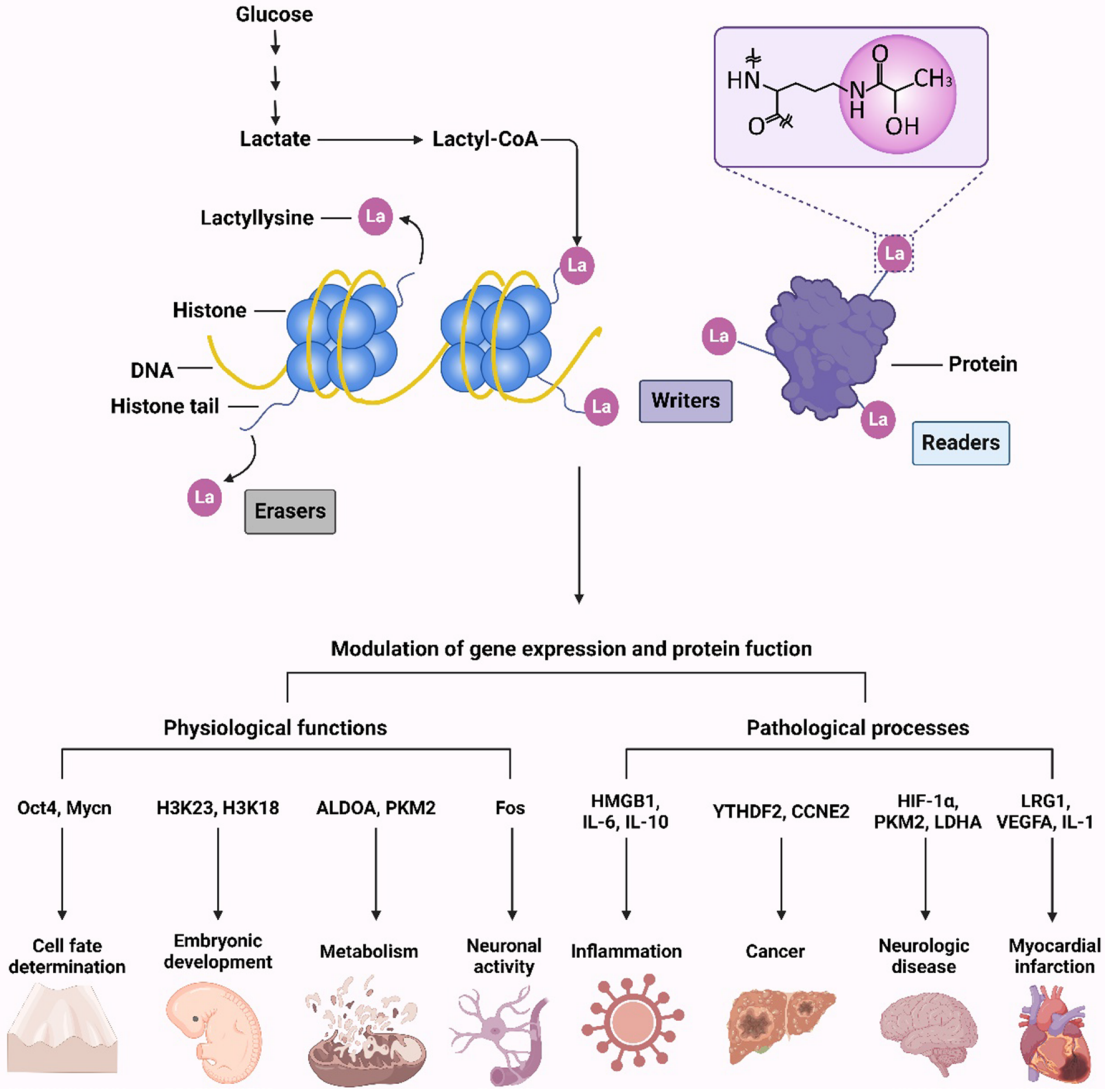
Protein lactylation is a new modification of posttranslational modification related to lactic acid which influences the physiological and pathological processes by regulating the gene expression and protein function

Microscopic molecules generated during cellular metabolism not only serve as metabolic intermediates but also act as signaling molecules that regulate various cellular activities. One significant mode of regulation involves the covalent modification of histones, wherein small molecules participate in epigenetic regulation [14, 15]. For instance, acetyl coenzyme A, produced during the tricarboxylic acid cycle, can acetylate lysine residues on histones. Notably, in a groundbreaking study conducted by Zhang et al. in 2019, lactate was identified as a novel molecule involved in a distinct histone modification known as histone lysine lactylation, which plays a role in gene expression regulation in macrophages. It has been reported that lactic acid can be covalently attached to histone lysine residues, thereby paving the way for further investigations into protein lactate modification [16]. This thesis aims to provide an overview of the mechanisms and functional implications of protein lactylation modification (as depicted in Fig. 1). The review highlights an overview of the discovery of lysine lactylation, outlines the potential "writers" and "erasers" responsible for protein lactylation, and discusses the diverse physiological and pathological roles of lactylation, further providing new insights into disease mechanisms and potential therapeutic approaches.

## The discovery of protein lactylation modification

In 2019 and 2020, two separate research teams led by Zhao et al. and Galligan et al. made significant strides in the field of protein lactylation (Kla), an emerging posttranslational modification [16, 17]. Zhao et al. utilized mass spectrometry techniques to detect a mass shift of 72.021 Da on-lysine residues of histones, thus unveiling the widespread occurrence of histone lysine lactylation through isotope metabolic labeling methods. Following this discovery, Galligan et al. further demonstrated that proteins are also susceptible to lactic acid modifications. These two research groups delved into various facets of this modification, including its characteristics, the sources of substrates, and the specific target proteins involved.

Zhao et al. postulated that lactylation modification is an actively enzymatic posttranslational process reliant on lactoyl coenzyme A (lactyl-CoA) as a substrate. Recently, the presence of lactoyl-CoA has been confirmed in mammalian cells and tissues using liquid chromatography–tandem mass spectrometry [18] (Fig. 1). In contrast, Galligan et al. suggested that lactylation modification is a passive, non-enzymatic acyl transfer process that employs lactoyl glutathione (LGSH) as a substrate. They also emphasized the role of glyoxalase II (GLO2) in regulating LGSH levels within cells. Additionally, Zhao et al. research primarily focused on lactylation modifications occurring on histones, revealing that histone lactylation represents a novel form of epigenetic regulation with profound implications for gene transcription. Conversely, Galligan et al. discovered lactylation modifications on several metabolic enzymes, indicating that lactylation of these enzymes can provide negative feedback regulation on the glycolytic pathway. Their study underscored the widespread presence of lactylation in human tissues and cells, emphasizing its regulatory impact on proteins beyond histones. In subsequent study, Zhao et al. identified potential “eraser” for lactylation, such as HDAC1 and HDAC2 [19]. Furthermore, latest studies have demonstrated that lactylation modification of proteins and histones is accomplished by enzymes, such as the p300 which the writer of lactylation, histone lactylation was significantly impaired upon p300 knockdown in mouse bone marrow-derived macrophages [20]. Therefore, as we see, the process is mostly an enzymatic reaction, but the modification exists not only in histone lysine but also in non-histone proteins.

Despite ongoing debates within the scientific community, the discovery of protein lactylation has not only expanded the horizon of research on posttranslational protein modifications but has also yielded valuable insights into the molecular mechanisms underpinning lactate’s role in critical physiological and pathological processes, including cancer, metabolism, and immunity. This groundbreaking revelation holds the potential to open up new avenues of exploration and deepen our comprehension of the multifaceted roles of lactate in diverse biological contexts.

## Identification of sites involved in protein lactylation modification

### Histone lactylation sites

**Fig. 2:**
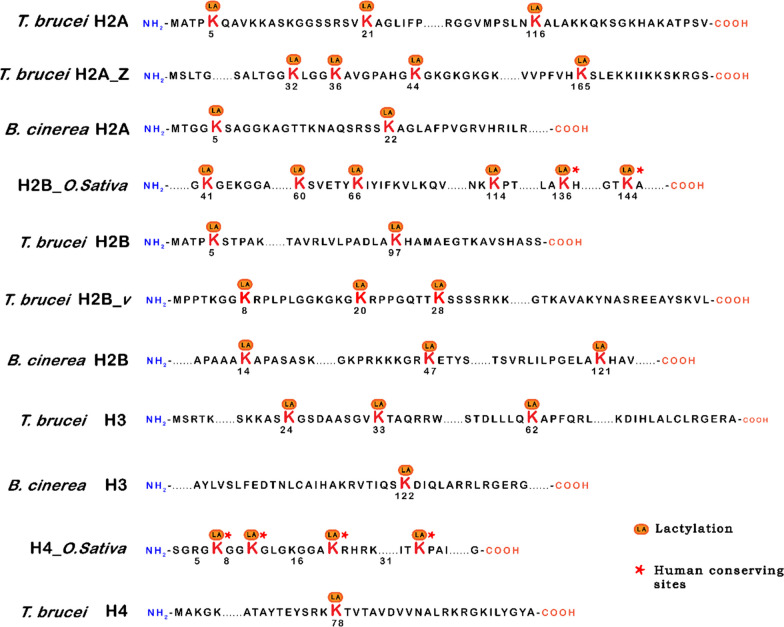
Lactylation modification in histone. Numbers represent amino acid positions of lactylation. The LA is the site of lactylation in lysine (K). The star means the site is also the lactylation in human histone

Histones are central players in chromatin structure, the intricate assembly of DNA and proteins that governs genome organization and regulation. Enzymes within cells can modify histone proteins through various epigenetic mechanisms, such as methylation [21–23], acetylation [24–26], and ubiquitination [27]. These modifications wield substantial influence over gene expression, DNA replication, and DNA repair processes. A significant advancement in our understanding of histone biology comes from the work of Zhao et al. They identified 26 histone lysine lactylation sites in HeLa cells and 18 sites in mouse bone marrow-derived macrophages. Further studies have expanded our insights into histone lactylation, revealing 16 sites in Trypanosoma brucei [28], 6 sites in Botrytis cinerea [29], and 14 sites in rice [30]. It is important to note that variations in the identified histone lactylation sites may arise from species-specific or spatiotemporal dynamics of lactylation (as depicted in Fig. 2). Crucial questions remain unanswered, including the identification of specific chromosomal regions enriched with histone lactylation sites and the elucidation of biological processes linked to these modifications. In-depth investigations are warranted to unveil these aspects and fully comprehend the significance of histone lactylation.

### Non-histone lactylation sites

**Fig. 3:**
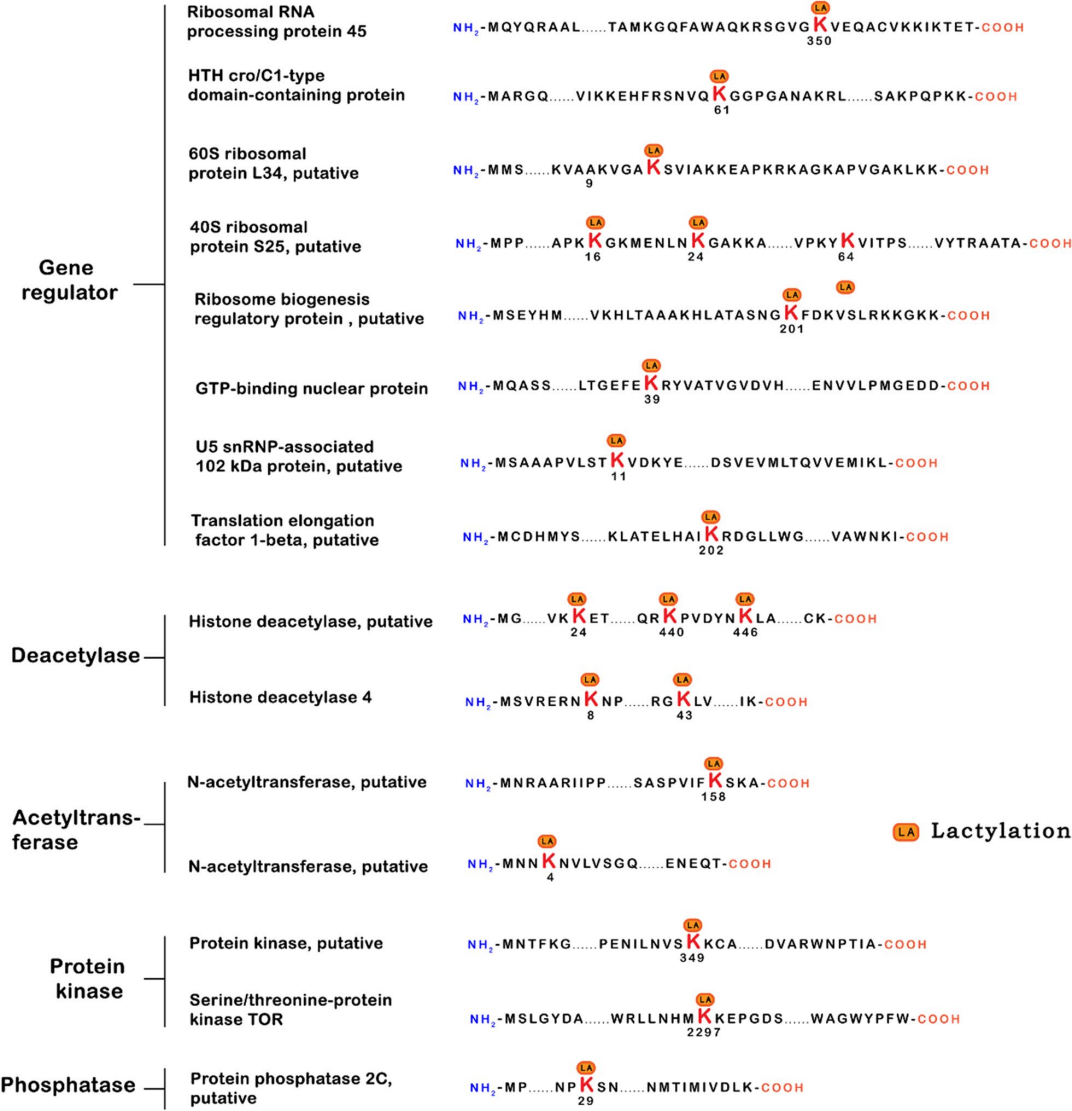
Lactylation modification in protein. Numbers represent amino acid positions of lactylation. The LA is the site of lactylation in lysine (K)

Non-histone protein modifications play pivotal roles in diverse cellular processes related to physiology and disease. These processes encompass gene transcription, DNA damage repair, cell division, signal transduction, protein folding, autophagy, and metabolism. Of particular significance, Gaffney et al. identified 350 lactic acid-modified proteins in HEK293T cells, focusing primarily on glycolysis and carbon metabolism pathways [17]. Simultaneously, research groups in Japan uncovered 63 lactic acid-modified proteins in the mouse brain, with 12 proteins exhibiting altered lactylation levels in response to social defeat stress. In the context of Botrytis cinerea [29], 273 lactylation sites within 166 proteins were identified, including 43 ribosomal structural proteins, hinting at a potential role for lactylation in protein translation. Moreover, Zhang et al. [28] identified 387 lactylation sites on 257 proteins in Trypanosoma brucei, with heat-shock protein 90 alone harboring 14 lysine residues susceptible to lactylation (as depicted in Fig. 3).

**Fig. 4:**
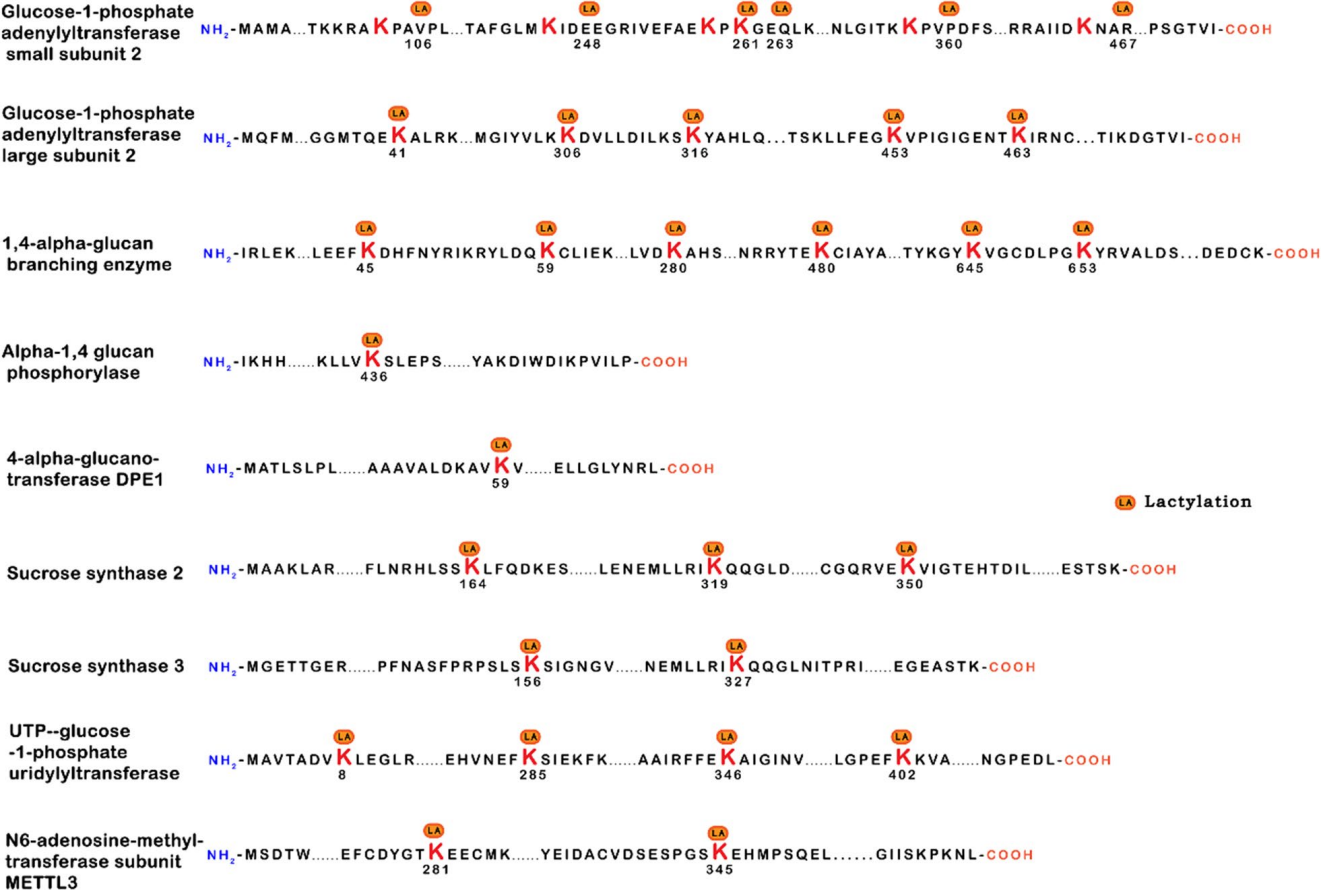
Lactylation modification in protein. Numbers represent amino acid positions of lactylation. The LA is the site of lactylation in lysine (K)

Lastly, extensive studies on developing rice grains unveiled 638 lysine lactylation modification sites distributed among 342 proteins [30]. These collective findings shed light on the prevalence and diversity of lactic acid-mediated protein lactylation across various biological systems, paving the way for further investigations into the functional implications of this intriguing posttranslational modification. Furthermore, in-depth examinations of lactylation modification and its regulation on individual proteins have yielded substantial insights. For instance, Xiong et al. [31] identified lactylation at lysine residues 281 and 345 of methyltransferase 3 (METTL3) in HEK293T cells, enhancing its affinity for N6-methyladenine (m6A)-modified RNA. Notably, lactylation has been observed in a diverse array of proteins located in the nucleus, cytoplasm, mitochondria, endoplasmic reticulum, and cell membrane [31, 32], suggesting that protein lactylation may play a role in regulating various biological activities (as depicted in Fig. 4). These observations underscore the broad impact of lactylation as a posttranslational modification and highlight its potential involvement in diverse cellular processes.

## Protein lactylation modification “writers” and “erasers”

Lysine acylation, a widespread and evolutionarily conserved form of protein posttranslational modification, exhibits dynamic spatiotemporal patterns [15]. While Gaffney et al.'s research has made significant contributions to this field, it is important to note that Galligan et al. [17] proposed an alternative perspective. They suggested that lactoyl glutathione (LGSH) could act as a donor for lactoyl groups in protein lactylation, and this process might occur independently of specific enzymes. This was demonstrated through the detection of lysine lactylation following the co-incubation of histone H4 and LGSH.

However, unlike the simplicity suggested by Galligan et al. many lysine acylation modifications require specific “writers” and “erasers.” These writers and erasers are enzymes responsible for adding or removing acyl groups, respectively. Histone acetyltransferases (HATs) and histone deacetylases (HDACs), well known for their roles in adding and removing acetyl groups from lysine residues, have also been implicated in the dynamic regulation of protein lactylation. Studies have highlighted the involvement of HATs and HDACs in controlling protein lactylation dynamics.

### “Writers” in protein lactylation modification

Among the “writers” involved in protein lactylation, the histone acetyltransferase p300 (p300) stands out. P300 is known for its role in inducing lysine acetylation of histone H3 to activate gene transcription. Recent studies have revealed that p300 is also involved in histone lactylation modification. For instance, overexpression of p300 in HEK293T cells led to increased levels of histone lactylation. Conversely, knocking down p300 in HCT116 cells and HEK293T cells resulted in decreased levels of H3K18la [16]. Additionally, lactate-induced histone lactylation was significantly impaired upon p300 knockdown in mouse bone marrow-derived macrophages [20]. Another study demonstrated that lactate induces lactylation of HMGB1 in RAW 264.7 cells, which can be antagonized by c646, an inhibitor of p300/CBP. Knockdown of p300 or CBP also significantly inhibited this phenomenon [32]. In mouse GV oocytes, overexpression of Tfap2a led to increased expression of p300 and elevated levels of pan-histone lysine acetylation (H4K12ac, H4K16ac) as well as pan-histone lysine lactylation (H3K18la, H4K12la) [33]. These experiments suggest that p300 and CBP may function as “writers” of lactylation modification, potentially acting independently or in concert to achieve lactylation of specific proteins. Furthermore, in *Escherichia coli*, Zhang et al. [34] demonstrated the widespread distribution of lysine lactylation (Kla) in bacterial proteins using Kla pan-antibodies. They identified YiaC as a lactate enzyme (writer) capable of catalyzing the addition of lysine lactate in vitro and in cells, thereby impacting the activity of metabolic enzymes. The latest research reported KAT8, a lysine acetyltransferase that acted as a pan-Kla writer, to lactylate eEF1A2K408 resulted in boosted translation elongation and enhanced protein synthesis which contributed to tumorigenesis in colorectal cancer [35]. These findings provide valuable insights into the involvement of p300, CBP, YiaC, and KAT8, as potential regulators of protein lactylation in diverse biological contexts.

### “Erasers” in protein lactylation modification

In a significant development, Zhao et al. (2022) [19] reported the identification of key "eraser" proteins involved in lysine lactosylation modification. They found that class I histone deacetylases (HDAC1-3) and class III histone deacetylases (SIRT1-3) were particularly effective in delactylation modification. In vitro experiments revealed that HDAC1 and HDAC3 played a role in reducing lactylation levels at H4K5 in HeLa cells. This discovery sheds light on the potential involvement of specific enzymes in regulating lysine lactylation. Furthermore, Zhang et al. discovered La-CoA as the donor of Kla in *Escherichia coli*, with YdiF identified as the lactoacyl-CoA-producing transferase responsible for converting lactic acid to lactoacyl-CoA. However, despite these findings, the precise enzymes and mechanisms involved in lactylation regulation in cells remain unknown, highlighting the need for further research in this area.

## Physiological functions of protein lactylation

### Lactylation and cell fate determination

Cell fate determination as the fundamental biological activity is the focus of recent research [36]. It is a complex process regulated at multiple levels, including the transcriptome, epigenome, and metabolome [37]. In mouse somatic cell reprogramming, the maternal transcription factor Glis1 plays a pivotal role by orchestrating a metabolic shift. Glis1 activates glycolytic genes while repressing somatic gene expression, resulting in robust lactate production. This increase in lactate production leads to elevated histone lactylation modification (H3K18la) and the expression of pluripotency genes. As a result, reprogramming efficiency is enhanced, even allowing for the reprogramming of senescent cells. This groundbreaking study introduces the concept of an “epigenome–metabolome–epigenome” cross-talk cascade regulated by Glis1 in pluripotent stem cell fate determination [38]. Additionally, in vitro cultures of mouse embryonic stem cells (ESCs) treated with lactate have shown the promotion of H3K18la modification in the promoter regions of genes related to germline and cleavage stage embryos, facilitating their widespread expression [39]. Notably, H3K18la has been implicated in regulating pluripotency genes like Oct4 and Mycn during somatic cell reprogramming. However, lactate treatment did not significantly alter the expression of other pluripotency genes, such as Oct4 and Sox2, in mouse ESCs [39], raising the need for further investigation to clarify this discrepancy. Periodontal ligament stem cells are important mesenchymal stem cells contributing to regenerating lost periodontal tissues and repairing bone defects. The study showed that Scopolamine promotes the lactylation of RUNX2 at the K176 site enhances the protein stability to promote the osteogenic differentiation of PDLSCs [40].

### Lactylation and embryonic development

Histone posttranslational modifications play a crucial role in epigenetic reprogramming during the transition from post-fertilization to preimplantation stages. Among these modifications, histone lactylation has emerged as a significant player in this dynamic process [41]. Yang et al. conducted a study revealing the temporal dynamics of histone lactylation in preimplantation embryos. In GV-stage oocytes, it was discovered that H3K23la, H3K18la, and pan-histone Kla were abundant. Additionally, MII-stage oocytes showed the presence of H3K23la and pan-histone Kla; however, H3K18la was not detected. Furthermore, histone lactylation was downregulated in zygotes following fertilization [42]. Whereafter, they revealed lactate induces endometrial H3K18la, which facilitates the implantation of embryos by remolding the endometrium [43]. Studies in pregnant sheep revealed that H3K18la levels in the endometrium increased in successful pregnancies but decreased in failed pregnancies. This suggests that lactate, produced through glycolysis in the maternal body, can promote histone lactylation in the endometrium via the maternal–fetal interface. This epigenetic regulation, in turn, influences gene expression related to redox homeostasis and apoptosis, shaping endometrial receptivity to embryos and promoting successful embryo implantation. Lactate could promote H3K18la, which facilitates transcriptional elongation of target genes in embryonic stem cells [39]. Therefore, it is illustrated that H3K18la may be involved in histone promoter activation and tissue-specific activity, as well as play a critical role in embryonic development and formation [44].

### Lactylation and metabolism

Metabolism regulates glycolysis, OXPHOS, and other metabolic pathways by modulating gene expression and signaling pathways [45]. The discovery of lactylation offers a new perspective for studying lactate metabolism mechanisms [46]. The discovery of lactylation as a phenomenon stem from observations of its occurrence in response to enhanced glycolysis and increased lactate levels. Notably, lactylation was found to regulate glycolysis by modifying enzymes like ALDOA and PKM2. Ye et al. demonstrated that lactylation modification of the metabolic enzyme ALDOA, conserved across various human tumor cell lines, significantly reduced its activity [47]. This discovery revealed a feedback regulatory mechanism where metabolic enzymes in the upper part of the glycolytic pathway are covalently modified to inhibit glycolytic activity following lactic acid accumulation, complementing the existing “end product inhibition” model in biochemistry. Moreover, in LPS-induced macrophage cells, lactate was shown to enhance the activity of pyruvate kinase by increasing lactylation at K62 of PKM2. This promoted the transition of macrophages to a repair phenotype under LPS-induced conditions [48].

### Lactylation and neuronal activity

Lactate, a vital molecule in the brain, serves multiple crucial functions in physiological activities [49]. The Kla of histone is widely distributed and regulates transcriptome remodeling in the human brain extensively during neural development. Dai et al. discovered that Kla may be present in brain nerve cells [50]. A study conducted by Hagihara H et al. [51] revealed that neuronal excitement and social defeat stress elevate lactate levels and protein lactylation levels in brain cells. They identified 12 protein lactylation events related to social defeat stress, including histone H1. Interestingly, the upregulated protein lactylation was positively correlated with the expression level of Fos proto-oncogene, a marker of neuronal activity, as well as with the observed decrease in social behaviors and increase in anxiety-like behaviors in stressed mice. This research not only confirms protein lactylation in the brain but also highlights its potential significance in regulating neuronal activity. Moreover, a study identified 63 candidate lactylated proteins in the brain. When lactate is added and neuronal excitation is induced, Kla levels could increase, but functional consequences have yet to be investigated [51]. Evidence suggests that neurons might take up astrocyte-derived lactate, which can affect Kla [52]. Proton-linked monocarboxylate transporters allow lactate to enter the plasma membrane. The process might be impacted by the intracellular lactate concentration and pH gradient, with lactate concentration playing a more important role. Neurons mainly absorb lactate and stimulate Kla [53, 54]. It has confirmed that neuronal excitation can affect brain function by increasing the immunoreactivity of Kla through lactate. However, the mechanism of action of lactate and its associated protein lactylation in the brain should be investigated in greater depth.

## Protein lactylation in pathological processes

### Lactylation and inflammation, immune, fibrosis

Once regarded as a metabolic waste product, lactate has emerged as a pivotal regulator of immune cells [10–12]. A groundbreaking study by Zhao et al. [16] revealed histone lysine residue modification through lactylation, opening new avenues to explore the non-metabolic functions of lactate in processes like infection and immunity. They showed that elevated Kla results in higher Arg1 and that this is associated with the development of a macrophage repair phenotype. Macrophages, essential in immune responses, are categorized as M1 (proinflammatory) and M2 (anti-inflammatory) types. The glycolytic pathway is highly active in M1 macrophages [55]. Studies show that toll-like receptors (TLRs)-mediated activation of downstream transcription factors and the synthesis of inflammatory components can be inhibited by PI3K/Akt [56]. BCAP is a crucial protein that connects TLR signaling to the PI3K/Akt pathway and functions as a junctional protein for PI3K signaling by B cells [57, 58]. Research has demonstrated that the lack of BCAP prevents the activation of the PI3K/Akt pathway, which in turn reduces aerobic glycolysis in cells. This, in turn, prevents the production of lactate and the modification of histones by lactylation, which further prevents macrophages from adopting the M2 phenotype linked to tissue repair [59]. Consequently, BCAP functions as an upstream bridging factor for aerobic glycolysis, facilitating the generation of lactate and stimulating lactylation of relevant histones produced by repair genes, thus contributing to the reduction of inflammation[60]. Notably, H3K18la levels in peripheral blood mononuclear (PBMC) from septic shock patients and histone lactylation in tumor-associated macrophages correlate positively with Arg1 transcript levels [16, 61], although recent research challenges the direct causal relationship between lactylation and Arg1 expression. However, a recent publication challenged the causal relationship between lactylation and Arg1 expression in bone marrow-derived macrophages stimulated with lipopolysaccharide. They found that Arg1 induction was dependent on autocrine paracrine interleukin 6 (IL-6) and IL-6 receptor, as well as signal transducer and activator of transcription 3 (STAT3), while lactylation was not directly involved [62]. Further studies are needed to clarify the regulatory relationship between histone lactylation and Arg1 gene expression in macrophages, considering the current controversy.

HMGB1, released by activated macrophages, plays a role in inflammation [63]. Clinical observations link lactate concentrations and HMGB1 levels in sepsis patients, though causality remains unclear. Studies reveal that macrophages uptake extracellular lactate, promoting HMGB1 lactation and acetylation via p300/CBP-dependent mechanisms. Modified HMGB1, with lactation/acetylation, is released from macrophages via exosomes, contributing to sepsis progression [32]. Elevated H3K18la levels in septic shock patients correlate with serum inflammatory factors, suggesting potential diagnostic and prognostic use [61]. The levels of H3K18la in septic shock patients were significantly correlated with serum levels of inflammatory factors such as IL-6 and IL-10. Consequently, H3K18la has the potential to serve as a diagnostic and prognostic biomarker for septic shock severity.

Histone lactylation also influences immune processes following myocardial infarction, promoting early activation of monocyte repair genes. This "metabolic–epigenetic–immune" cascade offers insights into enhancing cardiac repair post-myocardial infarction. Beyond bacterial infections, lactation plays a role in other inflammatory models [64]. For example, alveolar macrophages exhibit increased lactation levels during lung fibrosis [20]. Elevated lung myofibroblast lactate upregulates histone lactation in alveolar macrophages, promoting profibrotic gene expression. Inflammatory colitis models show that BCAP-deficient macrophages have reduced glycolysis, lactate content, histone lactylation, and injury repair gene expression, which can be rescued by exogenous lactate. Additionally, lactate-producing Saccharomyces cerevisiae reduces proinflammatory cytokines in macrophages, alleviating ulcerative colitis in mice. This involves lactate-induced enhancement of histone H3K18la. Furthermore, tumor-associated macrophages display elevated histone lactylation levels [65], suggesting its crucial role in modulating macrophage activation states during various inflammatory contexts. Comprehensive identification of genes regulated by histone lactylation in different inflammations requires further study.

### Lactylation and tumors

Abnormal energy metabolism is a recognized hallmark of tumors [66]. The Warburg effect, characterized by excessive lactate production by tumor cells, suggests that protein lactylation levels in tumors may also be significantly upregulated. Kla is a novel PTM generated from lactate that contributes significantly to the progression of tumors, typically by significantly altering the TME [67]. Indeed, Yu et al. [68] found that ocular melanoma samples showed higher levels of histone lactation modifications, particularly H3K18la, compared to normal samples. In ocular melanoma, they also discovered for the first time the interplay between histone Kla and m6A methylation. The presence of H3K18la was found to upregulate the expression of the m6A reader protein YTH N6-methyladenosine RNA-binding protein 2 (YTHDF2), which, in turn, accelerated the degradation of period circadian regulator 1 (PER1) and TP53 mRNA. This ultimately promoted the malignant phenotype of ocular melanoma cells [68]. In addition, anaplastic thyroid carcinoma (ATC) is a relatively uncommon but fatal kind of cancer [69]. A recent study revealed the interaction between ATC and Kla and discovered that aerobic glycolysis, which produces lactate in ATC cells, may enhance global protein Kla, including H4K12la, which activates numerous gene transcription important for ATC proliferation. Furthermore, BRAF V600E may affect H4K12la-driven gene transcription and cell cycle dysregulation by enhancing glycolytic flux [70]. In non-small cell lung cancer (NSCLC) cells, lactate downregulated glycolytic enzymes hexokinase 1 (HK-1) and pyruvate kinase (PKM) while upregulating tricarboxylic acid cycle enzymes succinate dehydrogenase A (SDHA) and isocitrate dehydrogenase 3γ (IDH3G) mRNA levels [71]. These effects preserved mitochondrial homeostasis while attenuating glycolysis. Interestingly, researchers discovered an enrichment of histone lactylation methylation in the promoter regions of both HK-1 and IDH3G. However, the specific conditions and underlying mechanisms by which histone lactylation modification promotes or represses gene transcription are yet to be fully elucidated. Moreover, the protein with lactylation modification also play an important role in cancer process. The K673 site of MRE11 protein is lactated by CBP to promote homologous recombination repair, which plays a key role in tumor chemotherapy resistance [72]. In cervical cancer, DCBLD1 with a lactylation site at K172 which can stabilize its expression, in order to inhibit G6PD autophagic degradation, activating pentose phosphate pathway (PPP) to promote the progression [73].

#### Lactylation in tumor microenvironment and immune cells

Lactylation modification has been observed in tumor cells and immune cells which infiltrating the tumor microenvironment (TME) [16, 31]. Specifically, histone lactylation levels were significantly higher in tumor-associated macrophages isolated from B16F10 melanoma and LLC1 lung carcinoma compared to peritoneal macrophages. Moreover, these lactylation levels positively correlated with the expression of the Arg1 gene, indicating an association between histone lactylation modification and the M2 phenotype of tumor-associated macrophages. The high levels of lactate and histone lactylation in macrophages may contribute to tumor formation and malignant progression. In hepatocellular carcinoma(HCC), decreased expression of sirt3 leads to lactylation modification of cyclin (CCNE2), thereby promoting tumor progression [74]. A study published in January 2023 in Nature Metabolism provided the first comprehensive map of lactylation modification in HCC, integrating proteome, lactylation modification, transcriptome, and genome analyses. This study demonstrated the significant role of lactylation in regulating tumor cell metabolism and suggested that lactylation acts as an "accelerator" of tumor metabolism, promoting the progression of HCC [75]. Gu et al. has illuminated that lactic acid can promote the lactylation modification of MOESIN at its K72, influencing the maturation and immunosuppressive capacities of regulatory Treg cells. Moreover, HCC patients who showed responsiveness to anti-PD-1 therapy exhibited reduced levels of MOESIN Kla modification in their Treg cells relative to non-responding patients [76]. In CRC cells, tumor-derived lactate fuels H3K18 lactylation to prohibit RARγ gene transcription in macrophages, consequently enhancing IL-6 levels in the TME and endowing macrophages with tumor-promoting functions [77]. Notably, Zhu et al. found that the pivotal function of Kla in modulating cellular plasticity and neuroendocrine differentiation within the context of prostate and lung adenocarcinomas. Mechanistically, a deficiency in the Numb/Parkin pathway increases lactate production, which increases histone Kla levels and stimulates transcription of neuroendocrine-associated genes [78]. Collectively, lactate-derived Kla significantly contributes to the complex interplay between cancer cells and the tumor microenvironment, thus facilitating tumorigenesis through various signaling pathways and cellular processes.

### Lactylation and other diseases

Additionally, Kla is implicated in a range of diseases. Research indicates that lactate produced by HK2 promotes H3K18la, thereby affecting the expression of metabolic-related genes. These findings suggest that targeting HK2 could be an effective strategy for treating liver fibrosis [79]. Moreover, Gao et al. have shown that MPC1 regulates the Kla of fatty acid synthase, and specifically, the Kla at the K673 site inhibits fatty acid synthase activity, leading to reduced liver lipid accumulation, which may offer insights into developing treatments for non-alcoholic fatty liver disease [80]. Alzheimer's disease (AD) stands out as the most prevalent neurodegenerative disorder characterized by the inflammatory activation of microglia and their metabolic reprogramming throughout the disease progression. In a study led by Pan et al. elevated levels of histone lactylation were initially identified in brain tissue samples from AD model mice and clinical AD patients [81]. Remarkably, this study pinpointed increased levels of H4K12la, primarily within the microglia surrounding amyloid plaques [82]. Importantly, the researchers found that H4K12la was enriched at the promoters of microglial glycolytic genes, particularly pyruvate kinase PKM2, thereby triggering their transcriptional activation. This activation led to an increase in glycolytic activity and lactate production, establishing a positive feedback regulatory loop termed the "glycolysis histone lactation-PKM2" loop. This loop further disrupts microglial homeostasis, fosters neuroinflammation, and contributes to the progression of AD. Encouragingly, targeted inhibition of PKM2 using small molecules like alkannin has displayed promising outcomes. Such inhibition significantly reduces the number of amyloid plaques in AD model mice and enhances their cognitive capabilities. These findings underscore the potential therapeutic strategy of targeting the glycolysis histone lactation-PKM2 loop to alleviate the pathogenesis of AD and enhance cognitive function [39]. In myocardial infarction, high levels of lactate induced the EMT by activating the TGF-β/Smad2 pathway. Mechanistically, lactate could induce lactylation of the Snail1 by CBP/p300 [83]. Moreover, histone lactylation of HIF-1α targets, such as Bmp5, Trpc5, and Kit, promotes pulmonary artery smooth muscle cell proliferation in hypoxic pulmonary hypertension [84].

## Conclusions and future perspectives

The investigation of protein lactylation as a novel posttranslational modification holds paramount importance in uncovering its functional and regulatory mechanisms in physiological and pathological processes, providing invaluable insights into disease development. While research has illuminated the roles of lactylation in diverse areas such as cancer, AD, inflammation, fibrosis, stem cell maintenance, embryonic development, and neuromodulation (as summarized in Table 1), the field of protein lactylation research remains in its nascent stages, leaving several critical questions unanswered. Firstly, it is pivotal to ascertain whether protein lactylation is an inevitable consequence of high lactate concentrations or if it undergoes precise regulation in a spatiotemporally controlled manner. Secondly, the direct substrates of protein lactylation, including lactoyl-CoA or lactoyl glutathione, their intracellular concentrations, and the enzymes responsible for their production from lactate, need identification. Thirdly, p300, CBP, YiaC, KAT8 and so on were showed the function of lactylation modifications. But the proteins involved in "writing," "reading," and "erasing" protein lactylation modifications require further exploration. Fourthly, in posttranslational modifications, the site of lactylation may replace ubiquitination to stabilize the expression [39]. Therefore, understanding the cross talk between histone lactylation modifications and other posttranslational modifications, including acetylation modifications, and their coordinated regulation of transcription and protein stability are essential. Lastly, apart from histones, it is crucial to identify other proteins regulated by lactylation. The establishment of comprehensive lactylation maps in various systems by proteomics and modification omics, particularly in human and mouse cells, would substantially advance the field of protein lactylation research, enhancing our comprehension of its functions. The discovery of protein lactylation modification has introduced fresh biological and functional perspectives to the role of lactate. In TME, the production, circulation, and utilization of lactate put a crucial role of tumors in both energy metabolism and epigenetic modification to facilitate tumor onset and progression. Thus, targeted lactylation may be used to treat cancer, and targeted lactate production improves the tumor microenvironment, as well as preventing and improving lactylation modification to fight cancer. Therefore, drug discovery efforts targeting lactate metabolism and protein lactylation modification offer new prospects for treating various diseases, including tumors. The ongoing exploration of protein lactylation modification holds the promise of unveiling novel therapeutic strategies and expanding our understanding of its biological significance.

**Table 1:** Lactylation modification of target genes and the mechanism in diseases

Disease type	Site of modification/enzyme	Study type	Target gene	Mechanism	References
Inflammation	Histone pan lysine lactylation	Bone marrow-derived macrophages from mice model	Arg1, Klf4	BCAP promotes reparative macrophage transition through histone lactylation	[59]
Septic shock	H3K18	The PBMC of healthy volunteers and critically ill patients	Arg1	High H3K18la expression showed higher IL-2, IL-5, IL-6, IL-8, IL-10, IL-17, IFN-α levels	[61]
Sepsis	Histone pan lysine lactylation/p300/CBP	RAW 264.7 cells	HMGB1	The lactylated/acetylated HMGB1 was released from macrophages via exosome secretion which increases endothelium permeability	[32]
Ocular melanoma	H3K18/p300	Tissues and ocular melanoma cells	YTHDF2	The upregulated YTHDF2 by H3K18 lactylation and promoted oncogenesis through inhibition of TP53 and PER1	[68]
Non-small cell lung cancer	H4K8	BEAS-2B, A549, and H1299 cells	HK-1, IDH3G	Glycolytic enzymes (HK-1, PKM) and TCA cycle enzymes (SDHA, IDH3G) were, respectively, downregulated and upregulated by lactate, and increased histone lactylation in promoters of HK-1 and IDH3G	[71]
Tumor-infiltrating myeloid cells (TIMs)	H3K18	Murine bone marrow-derived macrophages	METTL3	Lactate accumulated in tumor microenvironment induced METTL3 upregulation in TIMs via H3K18 lactylation	[31]
Hepatocellular carcinoma (HCC)	K348/SIRT3	HuH7 cells	CCNE2	SIRT3 delactylated CCNE2 K348la and promoted HCC cell apoptosis and prevented HCC outgrowth in vivo	[74]
Hepatocellular carcinoma (HCC)	K28 (AK2), K413 (IDH2)	Patients’ tissues of hepatitis B virus-related HCC	AK2, IDH2	Lactylation at K28 inhibits the function of AK2, facilitating the proliferation and metastasis of HCC cells	[75]
Alzheimer’s disease (AD)	H4K12	Prefrontal cortex and hippocampus of mice and AD patients	HIF-1α, PKM, LDHA	The glycolysis/H4K12la/PKM2-positive feedback loop exacerbates microglial dysfunction in AD	[82]
Myocardial infarction	H3K18	Monocyte–macrophages	LRG1, VEGFA, IL-10	Histone lactylation facilitated transcription of LRG1, VEGFA, and IL-1, which favored a reparative environment through their anti-inflammatory and proangiogenic activities	[64]

## Data Availability

Data sharing is not applicable to this article as no new data were created or analyzed in this study.
